# Glycolytic activity in human immune cells: inter-individual variation and functional implications during health and diabetes

**DOI:** 10.1097/IN9.0000000000000008

**Published:** 2022-11-01

**Authors:** Frank Vrieling, Xanthe A. M. H. van Dierendonck, Martin Jaeger, Anna W. M. Janssen, Anneke Hijmans, Mihai G. Netea, Cees J. Tack, Rinke Stienstra

**Affiliations:** 1 Division of Human Nutrition and Health, Wageningen University, Wageningen, The Netherlands; 2 Department of Internal Medicine (463) and Radboud Institute for Molecular Life Sciences, Radboud University Medical Center, Nijmegen, The Netherlands; 3 Department for Genomics and Immunoregulation, Life and Medical Sciences Institute (LIMES), University of Bonn, Bonn, Germany

**Keywords:** glycolysis, human immune cell function, lactate

## Abstract

An increase in glucose uptake driving aerobic glycolysis is a robust hallmark of immune cell activation. The glycolytic response supports functional alterations of the innate immune cells including the production and release of cytokines. Large inter-individual differences in the magnitude of this cytokine response are known to exist. In addition, the presence of disease is known to impact on immune cell function. Whether variation in metabolic responses of immune cells exist between individuals during health or disease is currently unknown. Here, we explore inter-individual differences in the glycolytic rate of immune cells using lactate production as readout upon activation using a variety of different stimuli. Glycolytic responses are subsequently associated to functional immune cell responses in healthy humans. In addition, we determined the glycolytic rate of immune cells and its association with immune function using patients diagnosed with diabetes mellitus. Based on the relative increase in lactate production after activation, distinct clusters of low, intermediate, and high responders could be identified, illustrating the existence of variation in glycolytic responses in healthy subjects. Interestingly, the production of cytokines mirrored these high-, intermediate-, and low-lactate patterns after pathogenic stimulation. In patients with diabetes mellitus, a reduced correlation was found between lactate and cytokine production, specifically for IL-6. Furthermore, based on the relative increase in lactate production, variability in the glycolytic response was reduced compared to healthy subjects. In conclusion, our results show a specific association between the glycolytic rate and function in human immune cells after stimulation with different pathogens. In addition to demonstrating the existence of glycolytic variability and specificity depending on the type of stimulus, the association between glycolysis and function in innate immune cells is altered during the presence of diabetes.

## 1. Introduction

Intracellular metabolism has been increasingly recognized as a key regulator of immune cell function ^[1–3]^. This has been illustrated by the observation that a well-orchestrated immune response toward different pathogens is accompanied by specific metabolic rewiring in immune cells ^[4]^. In particular, a metabolic shift from oxidative phosphorylation to aerobic glycolysis, also known as the Warburg effect ^[5]^, is seen as a hallmark for immune cell activation, both in the innate ^[6–8]^ and adaptive arm ^[9–12]^ of host defense. In innate immune cells, the upregulation of aerobic glycolysis enables the cell to fuel key processes including differentiation and inflammatory activation including the production of cytokines ^[8,13]^. The end product of glycolysis is lactate that is subsequently secreted by the cell and is used as a readout of its glycolytic rate. The increase in glycolysis allows for both a rapid enhancement in adenosine triphosphate production ^[14]^, yet also enables immune cells to use TCA cycle intermediates that contribute to inflammatory signaling ^[15,16]^. Dependence on glucose metabolism is exemplified by pre-treating inflammatory macrophages with the glycolytic inhibitor 2-deoxyglucose (2-DG) leading to reduced expression and production of IL-1β ^[17–19]^. Interestingly, inhibition of glycolysis also reveals a certain specificity since the production of TNFα seems generally unaffected by glycolytic inhibition in murine macrophages ^[18,19]^. Treatment with 2-DG of human macrophages also differentially impacts on cytokine production further establishing an important role of glycolysis in controlling cytokine production ^[20,21]^.

Within human populations, large variations exist in the production of cytokines: one of the most important determinants of the host immune response. These variations are established by a variety of different host-related factors, including genetic predisposition, gut microbiome composition, and environmental factors ^[22–27]^. Whether variations exist in the metabolic capacity of immune cells to increase the glycolytic rate upon activation and what factors may influence glycolysis in immune cells remains to be investigated. Since metabolism is a crucial determinant of immune cell function, potential differences in aerobic glycolysis of innate immune cells would serve to explain variations in immune responses between individuals.

Metabolic disorders, such as diabetes mellitus, are often linked to dysregulated immune responses and a chronic, low-grade inflammatory state of the body ^[28]^. The chronic inflammatory state may increase cardiovascular risk on the one hand, while defective responsiveness to pathogens will result in a higher susceptibility to infection on the other hand ^[29–32]^. Whether the presence of diabetes impacts on immune cell metabolism thus ultimately altering immune cell function is unknown.

The majority of studies performed in the field of immunometabolism to date have focused on the effects of specific metabolites in murine systems. Although these studies clarify important molecular mechanisms underlying immunometabolic rewiring, findings are difficult to extrapolate to human cells. Until now, large-scale cohorts exploring the role of cellular metabolism in driving differential immune responses in humans have been lacking.

In the current study, we therefore set out to explore the importance of aerobic glycolysis for innate immune responses to different pathogen-associated molecular patterns (PAMPs) that are known to drive glycolysis in human cells ^[4,33]^. We used cohorts of healthy subjects and patients with type 1 diabetes mellitus (T1DM) part of the Human Functional Genomics Project, which aims to study inter-individual variations of immune responses in humans ^[34]^. Using lactate as a marker for glycolytic rate ^[35]^, we determined the relationship between glycolysis and function using secretion of important cytokines after ex vivo stimulation of circulating immune cells as a readout. We mapped inter-individual variations in glycolytic activation of monocytes, defined potential host factors contributing to glycolytic variation and finally determined its contribution to immune cell function. We also studied whether the association between glycolysis and immune cell function in healthy individuals was altered in patients with T1DM. Our results suggest specificity regarding both cytokines and PAMPs in the association between glycolysis and function, which is altered during diabetes.

## 2. Methods

### 2. 1. Study population

The study was performed using 2 well-characterized, independent cohorts which are both part of the HFGP (http://www.humanfunctionalgenomics.org/) and which were recruited simultaneously. The 50FG cohort is a population-based cohort that consists of 56 healthy individuals with a Western European background and is a subset of the 500FG cohort ^[34]^. The absence of diabetes in the 50FG cohort was based on self-report. The 300DM cohort consists of 239 patients with T1DM, selected from the outpatient clinic at the Radboud University Medical Centre, The Netherlands. The HFGP was approved by the Ethical Committee of Radboud University Nijmegen, The Netherlands (NL54214.091.15, 2015-1930 and NL42561.091.12, 2012-550). All participants gave written informed consent before participation and experiments were conducted according to the principles expressed in the Declaration of Helsinki.

### 2. 2. Isolation of PBMCs and in vitro stimulation

In each cohort, blood from the cubital vein of volunteers was collected in 10 mL EDTA tubes (Monoject, VWR, Amsterdam, The Netherlands) and the PBMC fraction was isolated by density centrifugation using Ficoll-Paque (GE Healthcare, Chicago, IL, USA), followed by 3 washes in phosphate-buffered saline. 5 × 10^5^ PBMCs in were plated in 96-well plates in 100 µL RPMI 1640 (Dutch modified, Thermo Fisher Scientific, Bleiswijk, The Netherlands) supplemented with gentamycin, l-glutamine and pyruvate. 100 µL of either RPMI, or RPMI containing LPS, Pam3Cys, heat-killed *Candida albicans* conidia or heat-killed *Staphylococcus aureus* was added to obtain a final concentration of 100 ng/mL LPS, 10 µg/mL Pam3Cys, or 1 × 10^6^ parts/mL of either *C. albicans* or *S. aureus*. Supernatants were collected after 24 hours and stored at −20°C until assayed.

### 2. 3. Enzyme-linked immunosorbent assays

In cell supernatants, IL-1β, IL-6, TNFα, IL-10, IL-8 and IL-1RA levels were measured using DuoSet sandwich Enzyme-linked immunosorbent assays (ELISA) kits (R&D systems, Minneapolis, MN, USA) according to manufacturer’s instructions.

### 2. 4. Lactate measurements

The produced concentrations of lactate were determined in cell supernatants using the conversion of lactate by lactate oxidase (Merck, Rahway, NJ, USA). The subsequent oxidation of the Amplex Red reagent (ThermoFisher Scientific, Bleiswijk, The Netherlands) to resorufin via HRP (ThermoFisher Scientific, Bleiswijk, The Netherlands) was measured as a fluorescent signal.

### 2. 5. Statistical analysis

Data analysis was performed using R v1.3.6 and RStudio v1.2.1335. Samples with incomplete measurements for cytokines and/or lactate were excluded before analysis. Delta lactate values were calculated by subtracting the baseline lactate production under control (RPMI) condition from the lactate induced by immune cell activation (if baseline measurement was available). Mixed effects models of 50FG cohort samples were fitted and analyzed using the lme4 (v1.1.23) ^[36]^ and lmerTest (v3.1.2) ^[37]^ packages. Models included individual cytokines as dependent variables, a random intercept for each subject and the following covariates: Time + Lactate + Monocyte fraction + Age + BMI + Gender. For 300DM cohort samples, individual multiple linear regression models were fitted for each cytokine using the *lm* function from the stats package (v.3.6.1) and included the following covariates: lactate + monocyte fraction + age + BMI + gender. Regression coefficient estimates (slopes) and associated *P* values were subsequently extracted from individual models and plotted. Regression coefficients of 50DG and 300DM models were directly compared by the *z*-test ^[38]^. To investigate inter-individual variation in lactate responses, individual slopes were extracted per subject from a model which specified Stimulation as predictor variable and delta lactate as dependent variable. Subsequent *k*-means clustering on extracted slopes was performed using the factoextra package (v1.0.7) ^[39]^. Comparisons depicted in boxplots were tested for significance by Tukey’s test with Holm correction. Where indicated, *P* values were adjusted for multiple testing by false discovery rate (FDR). Plots were generated using ggplot2 (v3.3.2) ^[40]^ and cowplot (v1.1.0) ^[41]^.

## 3. Results

**Figure 1.**
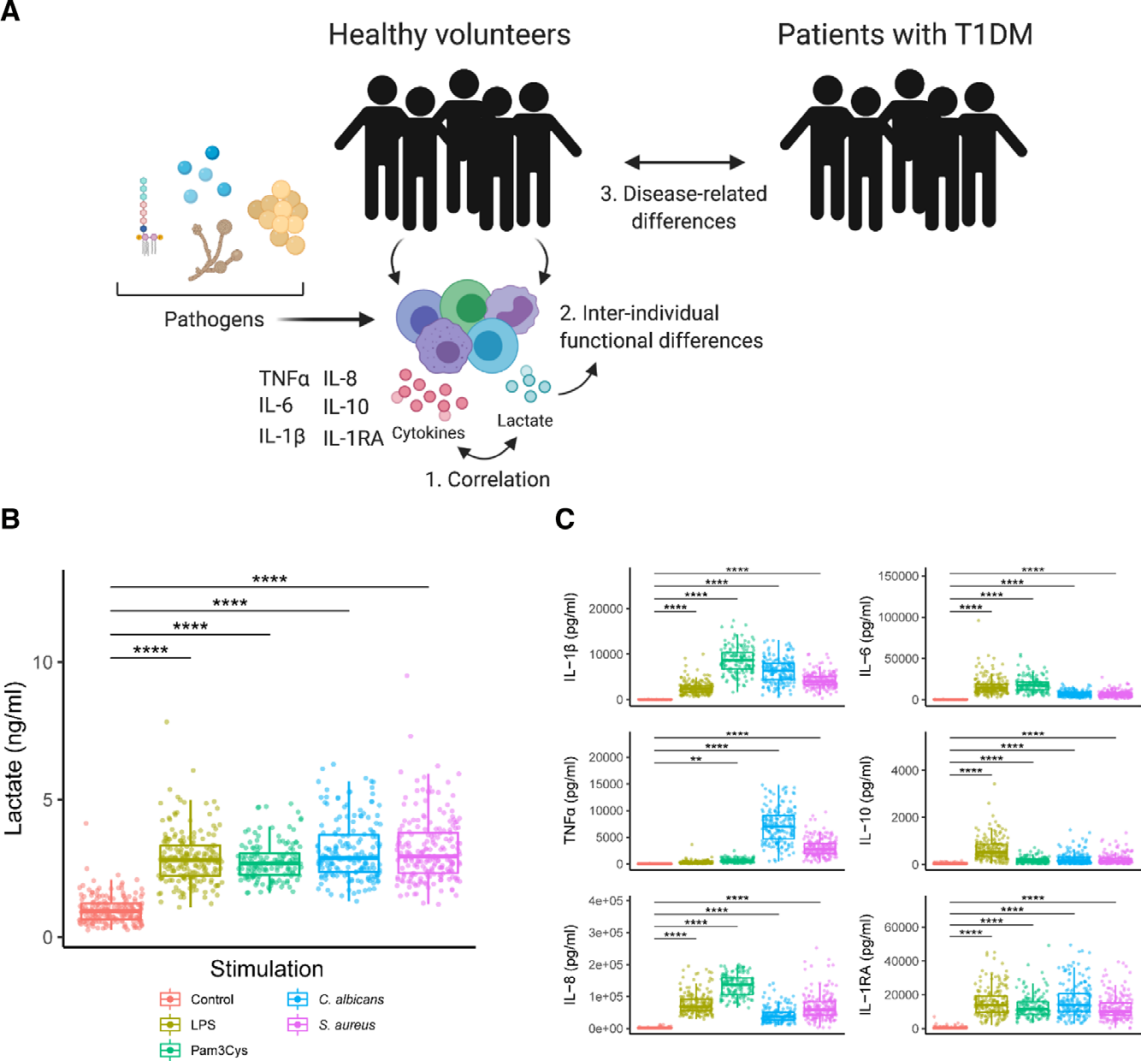
Overview of the study (A). PBMCs were isolated from healthy volunteers and patients with type 1 diabetes mellitus. After isolation, PBMCs were treated with RPMI (nontreated control), TLR-4 ligand LPS, TLR-2 ligand Pam3CysK4 (Pam3Cys), heat-killed *Staphylococcus aureus* or heat-killed *Candida albicans conidia* for 24 hours. The production of lactate was measured as a marker for aerobic glycolysis (B), and cytokines (C) (TNFα, IL-6, IL-1β, IL-8, IL-10, and IL-1RA) were determined as indication of the immune response. **P < 0.01, ****P < 0.0001. LPS, lipopolysaccharide; PBMNs, peripheral blood mononuclear cells; TLR, toll-like receptor.

Healthy volunteers and patients with T1DM were included in the study to explore normal variations in the correlation between aerobic glycolysis and the production of different cytokines in immune cells after inflammatory activation with different PAMPs. A schematic overview of the study setup is presented in Figure 1A. Detailed phenotypical characteristics of all study participants were collected (Supplementary Figure S1, http://links.lww.com/IN9/A0). Peripheral blood mononuclear cells (PBMCs) were isolated and stimulated with a variety of different PAMPs including the Toll-like receptor (TLR)-4 ligand lipopolysaccharide (LPS), TLR-2 ligand Pam3CysK4 (Pam3Cys), heat-killed *S. aureus* or heat-killed *C. albicans* for 24 hours to mimic a wide range of bacterial- or fungal-induced innate immune responses.

### 3. 1. Pathogenic stimulation leads to a robust increase in the production of lactate and cytokines

We first assessed the production of lactate after pathogenic stimulation. Although the production of lactate at baseline and after stimulation varied considerably both between stimuli and individuals, it robustly increased after all pathogenic stimulations in comparison to untreated cells (Figure 1B). This is in line with the well-established increase in the glycolytic rate of immune cells upon activation ^[5]^, which supports functional changes in the cells, including cytokine release ^[18,42]^. Indeed, the production of IL-1β, IL-6, TNFα, IL-10, IL-8, and IL-1RA was increased upon stimulation (Figure 1C), with each stimulus leading to a different pattern in cytokine secretion. The large range in the production of lactate and cytokines is indicative of significant inter-individual variation both in immune effector function and metabolism.

**Figure 2.**
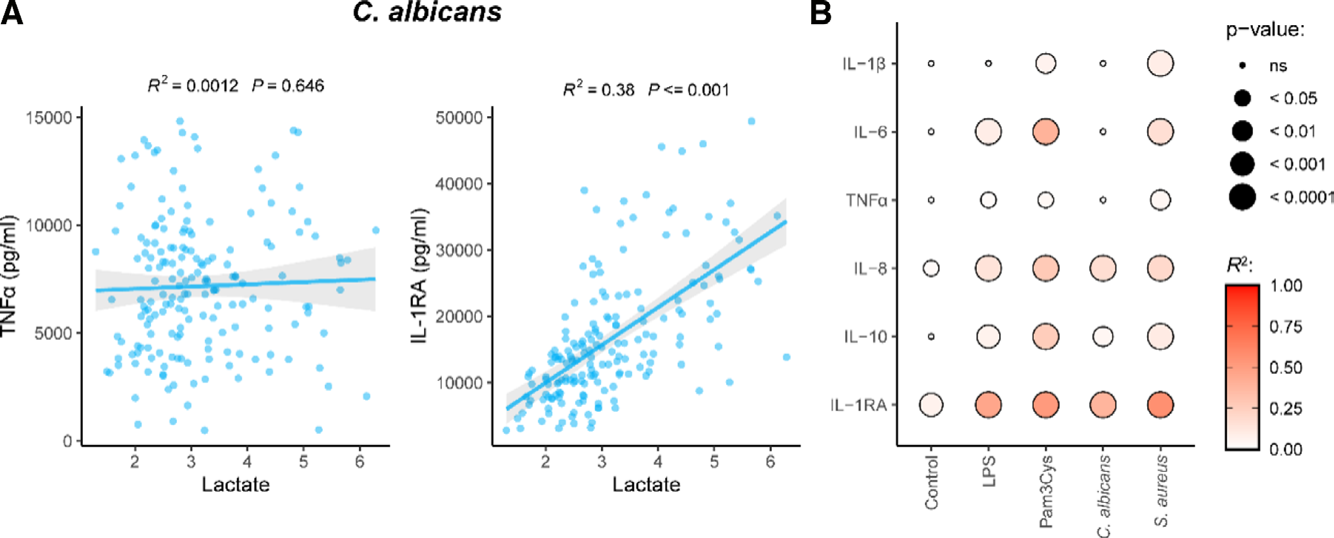
(A) Association between lactate and TNFα or IL-1RA after *C. albicans* stimulation. (B) *R*^2^ and *P* value of the correlation between the production of lactate and each cytokine after each stimulus. *P* values have been indicated in the figure panels. LPS, lipopolysaccharide.

Because glycolysis and immune cell function are closely intertwined, we next investigated the association between lactate and cytokine production for the different pathogenic stimuli. The results shown in Figure 2 indicate a differential association between lactate and cytokine production across different cytokines and stimulations. This is exemplified by the strong positive association between IL-1RA and lactate, whereas no association is observed between TNFα and lactate after *C. albicans* stimulation (Figure 2A). An overview of the associations between lactate and cytokine production observed after stimulation with different PAMPs is shown in Figure 2B. Several cytokines show a robust association with lactate including IL-8 and IL-1RA across different types of stimulations (Figure 2B). Also, different stimuli lead to specific associations between glycolysis and cytokine production, with Pam3Cys and *S. aureus* often eliciting the strongest associations between lactate and cytokine production. In contrast, upon *C. albicans* stimulation a lower association between lactate and cytokines is observed (Figure 2B). An overview of the *P* value, FDR and the coefficient of determination (*R*^2^) of the correlations between lactate and cytokines for all stimulations is presented in Supplementary Table S1, http://links.lww.com/IN9/A0.

### 3. 2. The production of lactate by PBMCs after pathogenic stimulation is affected by host characteristics

**Figure 3.**
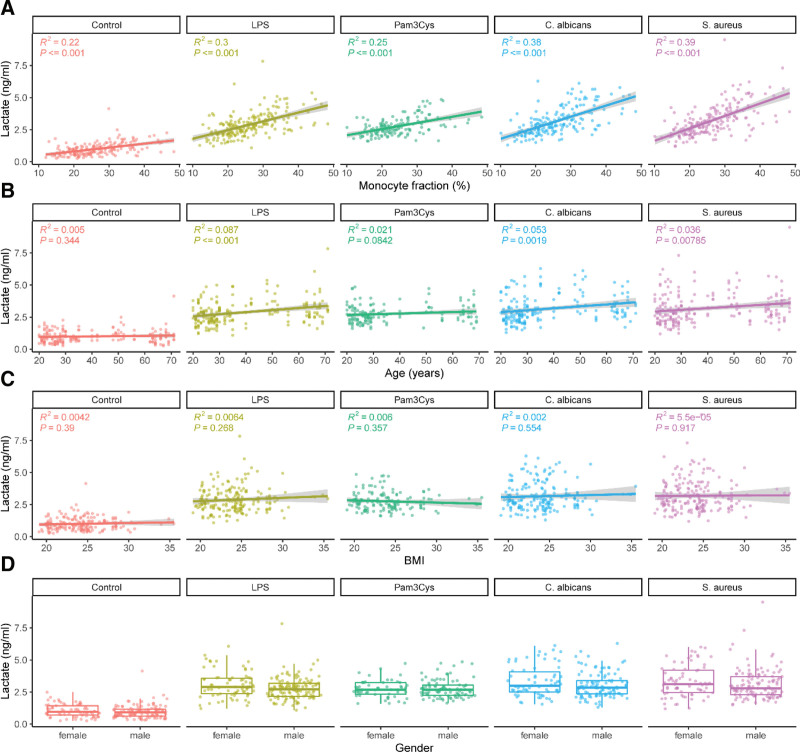
The associations between lactate production and host characteristics. PBMCs were isolated from healthy volunteers and treated with either untreated cells, LPS (100 ng/mL), Pam3Cys (10 µg/mL), *S. aureus* (1 × 10^6^/mL), or *C. albicans* (1 × 10^6^/mL) for 24 hours. The production of lactate was plotted against the percentage of monocytes (A), age (B), BMI (C), and gender (D). BMI, body mass index; LPS, lipopolysaccharide; RPMI, Roswell Park Memorial Institute medium.

Within the first 24 hours after stimulation, immune responses are predominantly driven by innate immune cells. Because the production of TNFα, IL-1β, IL-6, and IL-8 is mainly monocyte-specific, we assumed that monocytes were the cell type responsible for the majority of lactate and cytokines measured in the supernatants, especially after pathogenic stimulation of the cells. Variations in monocyte numbers in the PBMCs isolated from the study participants could therefore explain part of the variation in lactate production. Indeed, a linear regression analysis revealed that the percentage of monocytes was an important determinant of lactate production by PBMCs confirming their importance in lactate production upon innate immune cell stimulation (Figure 3A). This was particularly evident after stimulation of the cells reflecting the activation of monocytes part of the PBMCs.

Additionally, other host factors such as age, BMI, and sex known to determine host immune response ^[27]^ could also impact on the correlation between glycolysis and cytokine production. However, linear regression analysis revealed that age (Figure 3B), BMI (Figure 3C), and gender (Figure 3D) only have a minor impact on the glycolytic rate of the cells upon activation using the various stimuli.

### 3. 3. The correlation between lactate production and cytokine production is cytokine- and stimulus-specific

**Figure 4.**
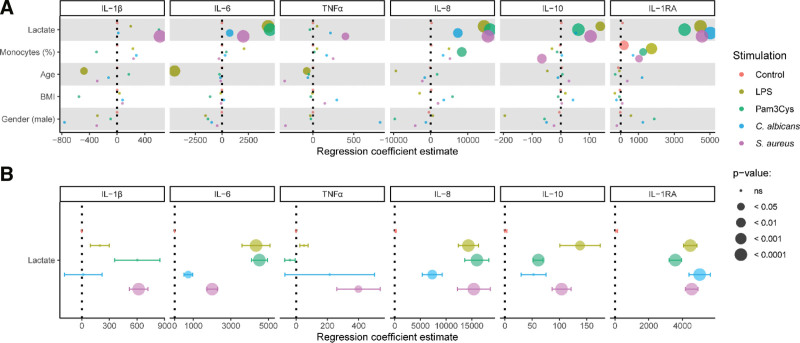
Increase of both lactate and cytokine secretion after pathogenic stimulation is predominantly cytokine-specific. PBMCs were isolated from healthy volunteers and treated with either RPMI (nontreated control), LPS (100 ng/mL), Pam3Cys (10 µg/mL), *S. aureus* (1 × 10^6^/mL), or *C. albicans* (1 × 10^6^/mL) for 24 hours. The relationship between lactate, host factors and cytokine secretion was explored by fitting separate mixed models for each combination of cytokine/stimulation, including percentage of monocytes, age, BMI, and sex as covariates (scaled) (A). The correlation between the production of lactate with the production of each cytokine was determined for all stimuli as the regression coefficient estimate (B). *P* values were adjusted for multiple testing by FDR correction and are shown in the figure panels. BMI, body mass index; LPS, lipopolysaccharide; RPMI, Roswell Park Memorial Institute medium.

To determine intrinsic differences in lactate and cytokine responses between individuals, separate mixed models were fitted for each cytokine-stimulation combination, for which host factors including the monocyte percentage were added as covariates in a stepwise fashion to evaluate their individual impact (Supplementary Figure S2, http://links.lww.com/IN9/A0. Because all of the included factors displayed some impact on the model parameters, they were used as covariates in the final model to account for their influence. Subsequently, regression coefficients (slopes) and associated *P* values were extracted for all covariates from each individual model to compare their relative contribution to the production of IL-1β, IL-6, TNFα, IL-10, IL-8, and IL-1RA. For all pathogenic stimuli, lactate production was by far the most relevant indicator for the production of several cytokines (Figure 4A). Other host factors sporadically displayed significant associations, such as an observed inverse relationship between age and production of IL-1β, IL-6, and TNFα after LPS stimulation, or the specific association between monocyte percentages and IL-1RA levels (Figure 4A).

We observed that lactate showed a significant positive correlation with the production of several cytokines for the majority of the pathogenic stimuli (Figure 4B). However, strength and significance of the correlation varied, depending on specific cytokines and stimuli. IL-6 responses are significantly correlated to lactate for all stimuli, with the strongest association for Pam3Cys and LPS. Of all cytokines, the production of TNFα was least significantly correlated to lactate production, only after stimulation with *S. aureus*. IL-10 production was significantly correlated to lactate production after all stimuli, except *C. albicans*, and the strongest correlation can be seen for LPS. The production of IL-8 was correlated to lactate production for all stimuli, with the strongest correlations for Pam3Cys, LPS, and *S. aureus*. Similarly, IL-1RA production was also significantly correlated to lactate production after all stimuli, with strong correlations found after each stimulus. *C. albicans* seems to be the stimulus that leads to the weakest correlations between cytokine and lactate production (Figure 4B). It appears that the relationship is partly cytokine-specific, with the strongest correlations for IL-6, IL-10, IL-8, and IL-1RA. On the other hand, the relationship between cytokine and lactate production also shows specificity for certain stimuli, where stimulation with *S. aureus* most often results in a significant correlations between glycolysis and cytokine release.

### 3.4 Clustering of subjects based on inter-individual differences in lactate response reveals the existence of high and low responders

**Figure 5.**
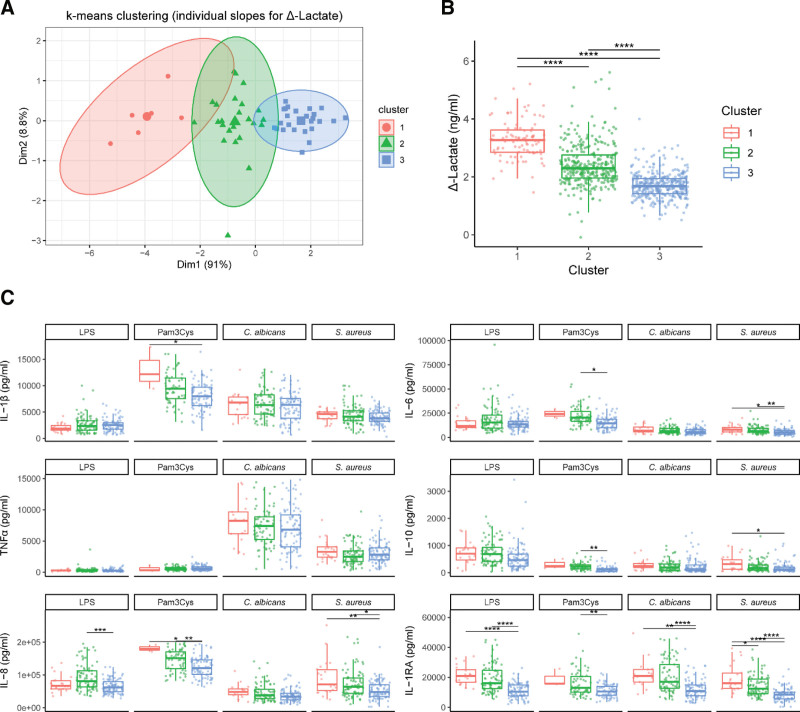
Clustering inter-individual lactate production reveals the existence of high and low responders. A random slope model was fitted on all samples, and individual slopes of the effect of stimulation on delta lactate production were extracted for each subject. Extracted slopes were subsequently used for *k*-means clustering of subjects into three clusters (A). The delta of the lactate response (B) and the production of IL-1β, IL-6, TNFα, IL-10, IL-8, and IL-1RA (C) was plotted for each cluster. *P < 0.05, **P < 0.01, ***P < 0.001, ****P < 0.0001. LPS, lipopolysaccharide.

The lactate concentrations (Figure 2A) show that lactate production of immune cells varies between individuals. To explore potential underlying causes and functional consequences, we aimed to identify group patterns of different glycolytic responders based on relative lactate responses after stimulation. Using *k*-means clustering, data were separated into three groups representing between-subject differences in lactate production after the different stimuli (Figure 5A) and identifying the existence of high (red), intermediate (green), and low (blue) responders in the relative production of lactate after pathogenic stimuli (Figure 5B).

When plotting the production of cytokines for each of the three clusters after treatment with different stimuli, the production of cytokines mirrored these high-, intermediate-, and low-lactate patterns after pathogenic stimulation (Figure 5C). Especially the differences in cytokine production between the high- and low-responding groups were distinct, with significant differences for IL-1RA after stimulation with LPS; for IL-1β and IL-8 after stimulation with Pam3Cys; for IL-1RA after stimulation with *C. albicans*; and for IL-6, IL-8, IL-10, and IL-RA after stimulation with *S. aureus*. Between the intermediate- and low-responding groups, differences were also well-defined, with significant differences for the production of IL-8 and IL-1RA after stimulation with LPS; for IL-6, IL-8, IL-10, and IL-1RA after stimulation with Pam3Cys; for Il-1RA after stimulation with *C. albicans*; and for IL-6, IL-8, and IL-1RA after stimulation with *S. aureus*. Differences between the high- and intermediate-responding groups were less evident, where only the production of IL-1RA was significantly different after stimulation with *S. aureus.*

Although host factors such as age, gender, and BMI were seen to play minimal roles in the association between lactate and cytokine production, we next assessed their relevance for the formation of the clusters reflecting relative lactate response. The formation of these clusters could not be explained by host factors that were accounted for in the model, although univariate plotting of host factors did still reveal the importance of monocyte frequency in these clusters, and revealed a nonsignificant skewing toward a lower age for the low-responding group (Supplementary Figure S3A http://links.lww.com/IN9/A0). Although the observed clusters seem to predict the magnitude of the functional response through the production of several cytokines, they likely suggest the existence of a wide range in the glycolytic rate in healthy individuals.

### 3.5 The relationships between cytokine and lactate production are altered in patients with T1DM compared with healthy subjects

**Figure 6.**
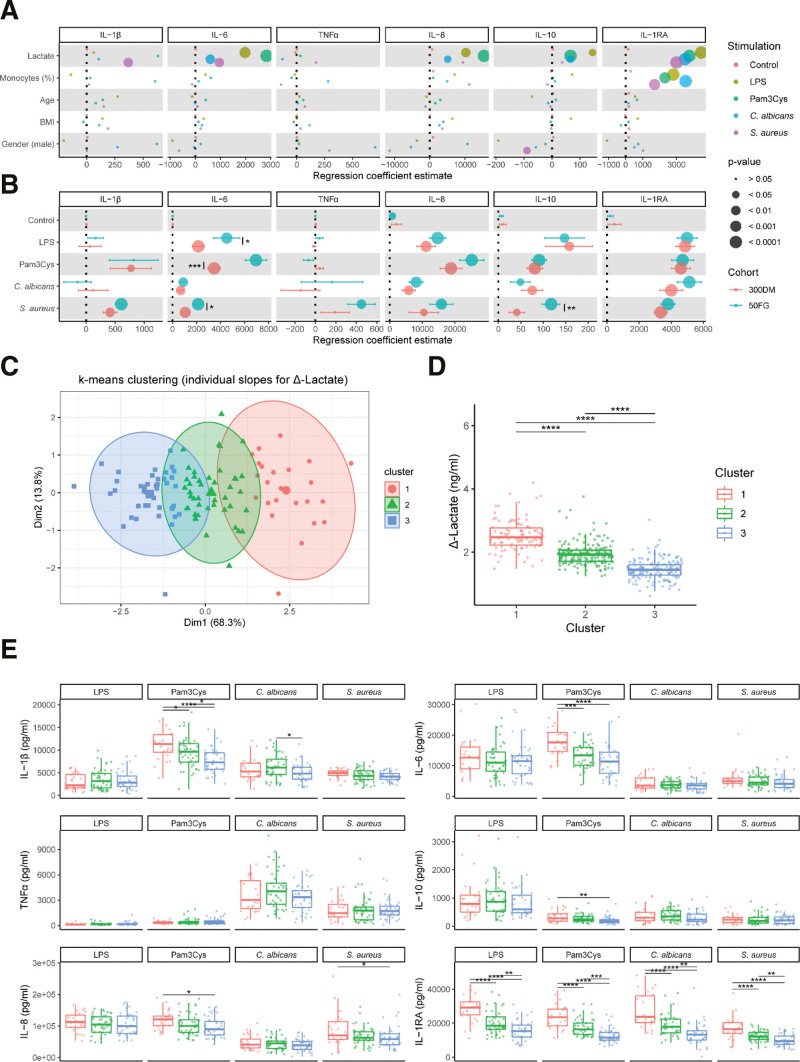
The relationship between subject characteristics or cytokine production and lactate production are altered in patients with T1DM compared with healthy subjects. PBMCs were isolated from patients with T1DM and treated with either RPMI (nontreated control), LPS (100 ng/mL), Pam3Cys (10 µg/mL), *S. aureus* (1 × 10^6^/mL), or *C. albicans* (1 × 10^6^/mL) for 24 h. The relationship between lactate, host factors, and cytokine secretion was explored by fitting separate multiple linear regression models for each combination of cytokine/stimulation, including percentage of monocytes, age, BMI, and sex as covariates (scaled) (A). From each individual model, the regression coefficient (slope) for lactate was extracted and directly compared with those extracted from the mixed model fitted for healthy subjects in Figure 4B (not scaled) (B). *K*-means clustering was performed for the individual slopes based on the delta of the lactate response for all subjects, corrected for percentage of monocytes, sex, BMI, and age (C) and the concentrations of lactate (D) and IL-1β, IL-6, TNFα, IL-10, IL-8, and IL-1RA (E) were plotted for each cluster. * *p* < 0.05, ** *p* < 0.01, *** *p* < 0.001, **** *p* < 0.0001. BMI, body mass index; LPS, lipopolysaccharide; PBMCs, peripheral blood mononuclear cells; RPMI, Roswell Park Memorial Institute medium; T1DM, type 1 diabetes mellitus.

Diabetes mellitus is a metabolic disease that is known to associate with aberrated functional innate immune responses and altered metabolic responses of the immune cells ^[43]^. Therefore, we explored whether the observed associations between lactate and cytokine production in healthy subjects were different in patients with T1DM. An overview of the *P* value, FDR, and the coefficient of determination (*R*^2^) of the correlations between lactate and cytokines for all stimulations is presented in Supplementary Table, http://links.lww.com/IN9/A0. The production of lactate was plotted against the percentage of monocytes, age, BMI, gender, diabetes duration, and HbA1c levels (Supplementary Figure S4, http://links.lww.com/IN9/A0). Next, these parameters were included as covariates in a multiple linear regression model for each combination of cytokine and stimulation as was done similarly in the healthy individuals. Regression coefficient estimates (slopes) and associated *P* values were subsequently extracted for each covariate (Figure 6A).

In patients with T1DM, we observed an inverse relationship between gender and IL-10 production after *S. aureus* stimulation compared to the healthy subjects. Also, the relationship between age and IL-1β, IL-6, or TNFα production was not seen in this population compared to the healthy subjects (Figure 6A), arguing that the presence of diabetes supersedes the impact of age.

When directly comparing the relationship between lactate and cytokine production in healthy subjects versus patient with T1DM (Figure 6B), it can be seen that the correlations observed for IL-10 or IL-6 and lactate production after stimulation with *S. aureus* were significantly attenuated and the correlation between lactate and TNFα disappeared (Figure 6B). Additionally, the relationship between IL-6 and lactate production was significantly weaker for patients with T1DM after Pam3Cys and LPS stimulation (Figure 6B). Based on these observations, the association between glycolytic metabolism and function of innate immune cells seems to be partly attenuated in patients with T1DM.

Using the same clustering approach leading to the identification of high, intermediate, and low responders in lactate production of healthy individuals, we identify similar groups in the patients with T1DM. Clustering led to the identification of distinct groups, differing in their relative lactate response to stimuli (Figure 6C and D). However, the three different clusters displayed a lower degree in variation compared with healthy subjects (Figure 5B). Nonetheless, similar patterns were found with univariate plotting of host factors, except for age (Supplementary Figure S3B, http://links.lww.com/IN9/A0).

When comparing the production of IL-1β, IL-6, TNFα, IL-10, IL-8, and IL-1RA among the three clusters, patterns were found to be generally comparable to the clustering observed in the healthy subjects, although differences were present for specific cytokines or stimuli. Indeed, several significant differences found between clusters in healthy subjects after treatment with *S. aureus* were not found in patients with T1DM, for instance, for the production of IL-6, IL-10, and IL-8 (Figure 6E). Similarly, several differences that were found in IL-8 production after LPS or Pam3Cys stimulation in the clusters based on healthy subjects, were not found to be significant in the clusters based on patients with T1DM (Figure 6E). In contrast, some of the T1DM-based clusters actually displayed stronger distinctions, exemplified by the response to Pam3Cys resulting in significant differences between each of the groups for the production of IL-1β, IL-6, and IL-1RA (Figure 6E). Furthermore, the differences between the high- and intermediate-responding groups seem more evident for IL-1RA after treatment with LPS or *C. albicans*, although there is a significant difference between the intermediate- and low-responding groups for the production of IL-1β after *C. albicans.* Overall, the clusters of high, intermediate, and low responders in T1DM show less distinct differences in the production of cytokines after treatment with *S. aureus*, yet more pronounced differences in the production of cytokines after treatment with Pam3Cys or *C. albicans*.

## 4. Discussion

In this study, we explored the link between the glycolytic response of stimulated immune cells and the functional response of the cells measured by the cytokine production capacity in humans. Collectively, our data demonstrate a robust link between aerobic glycolysis and cytokine production in immune cells upon stimulation during both health and the presence of diabetes. Stimulation of immune cells by Pam3Cys or *S. aureus* elicited the strongest relationships between lactate and cytokine production in healthy subjects and the production of IL-1RA was most significantly linked to lactate production for all pathogenic stimuli. Clustering analysis of relative lactate responses resulted in the identification of high, intermediate, and low responders. The identification of these groups most likely reflects the existence of glycolytic variation observed in immune responses of healthy individuals. However, in patients with T1DM, the correlation between lactate and cytokine production is altered. For example, the correlation between lactate and IL-6 production was weaker compared to healthy individuals. Although clustering of patients with T1DM also resulted in the identification of high, intermediate, and low responders based on lactate production, glycolytic variation was lower compared to healthy subjects. Altogether, our results show a specific association between the glycolytic rate and function in human immune cells after stimulation with different pathogens that is altered during the presence of diabetes.

Our observed correlations between lactate and cytokine production are confirmative of previously found correlations between immune cell metabolism and function that have mainly been assessed in cells from murine origin. Interestingly, the magnitude of this association seems to differ for various cytokines, but is also affected by the type of stimulus used to induce immune cell activation. The specificity regarding cytokines is in line with earlier reports from in vitro studies demonstrating attenuated cytokine release after treatment with 2-DG. These studies revealed that the production of some cytokines depends more on glycolysis compared to others. Interestingly, the production of IL-1β, IL-6, and IL-10, but not TNFα, could be inhibited by the addition of 2-DG ^[18,44]^, which is in line with the results of this study. Hence, TNFα showed only a weak correlation with lactate production for all stimuli tested, implying an apparent independence of TNFα from glycolytic regulation.

Since lactate is the end product of glycolysis, we have used lactate concentrations accumulating in the medium as a marker for the glycolytic rate. The robust correlations we observed between lactate and the number of monocytes present in the PBMCs strongly implies that it serves as a valid marker for the glycolytic rate of innate immune cells after activation. Also, the consistent increase in lactate concentration after stimulation of the cells known to induce an increase in glycolysis implies that lactate serves as a valid marker of long-term glycolytic activation of immune cells. In line with our work, several recent studies have confirmed a robust association between the rate of glycolysis and lactate levels ^[45–47]^.

Interestingly, the observed variation in lactate production implies large variations in glycolytic metabolism of immune cells in humans. Our analysis revealing the existence of low and high lactate producers suggests that the differences in aerobic glycolysis could partly explain the observed variations in immune responses between individuals. However, host-related factors responsible for the differences in glycolytic metabolism in immune cells remain unknown. To elucidate these factors, we used clustering analysis to identify high-, intermediate-, and low-responding individuals based on the delta of the lactate production after immune cell activation, reflective of their glycolytic flexibility. Within these clusters, we investigated whether different patterns could be found related to monocyte count, gender, BMI, or age. Whereas the effect of gender, BMI, or age did not seem to play an important role in the formation of the clusters, differences in monocyte numbers could be seen between the groups, which were also found in the uncorrected associations between lactate and cytokine prediction. This validates the assumption that a major fraction of the measured cytokines and lactate are indeed produced by monocytes. To be able to investigate the intrinsic differences between individuals, we accounted for monocyte frequencies by including it as covariate in the mixed models. However, although the production of lactate was found to be a better predictor for cytokine responses than the monocyte count in the mixed models, it thus proved difficult to fully correct for the differences in monocyte populations in PBMCs in the formation of the low-intermediate-high producers. Even after correcting for absolute monocyte count, a residual effect remains, which is potentially a biological inter-cellular amplifying effect driven by increased monocyte frequencies. Although using PBMC fractions better resembles the in vivo situation, and circumvents laborious isolation steps, to draw more specific conclusions it would be advisable to isolate single-cell populations to fully rule out the amplifying or dampening effects of variations in the monocyte count.

The cytokine that was most unambiguously associated with lactate production in this study was IL-1RA. IL-1RA functions as an anti-inflammatory cytokine through binding the IL-1 receptor (IL-1R1/IL-R2), thereby acting as a natural antagonist for both IL-1α and IL-1β in response to their production ^[48]^. IL-1β, even at low concentrations, is a strong inducer of IL-1RA that is known to inhibit IL-1 bioactivity, thus functioning as a negative feedback signal. Therefore, the higher levels of IL-1RA are often used as a surrogate marker for IL-1 activity. The consistent association of IL-1RA with lactate production could therefore indicate that its production and the production of IL-1 itself is highly dependent on aerobic glycolysis.

In the specific association between cytokines and aerobic glycolysis, relative kinetics should be taken into account as well. Cytokines such as IL-1β and TNFα are often upregulated relatively early in the response to pathogens, whereas the production of IL-1RA starts in response to IL-1β ^[48]^. Because both lactate and cytokines were measured after 24 hours, the correlation for cytokines could be different depending on their peak production time. This may partly underlie the stronger associations found for anti-inflammatory cytokines with lactate production, because they belong to the late responders. Additionally, lactate itself can also affect the production of certain cytokines and form a negative feedback loop to inhibit glycolysis ^[49–52]^. Lactate has also been shown to reduce the production of TNFα ^[51]^, which may partly explain the relative lack of association between lactate and TNFα production. In addition, lactate transporters present on immune cell might result in altered concentrations in the medium of the cells that would partly be independent of the cellular glycolytic rate.

The associations between lactate and cytokine production appear to be stimuli-specific. The observed differences could be explained by the different pathways activated by specific Toll-like agonists and pathogens (TLRs) ^[53]^. This is exemplified by the strong associations between lactate and cytokine production after stimulation with Pam3Cys and *S. aureus*. Both Pam3Cys and *S. aureus* are recognized through TLR-1/2 ^[54–56]^ indicating that the downstream pathways lead to upregulation of aerobic glycolysis and cytokine production serving to explain the positive associations. TLR-specificity is further illustrated by the association between IL-1β and lactate after Pam3Cys and *S. aureus* stimulation, whereas no significant association is observed after stimulation with the TLR-4 ligand LPS ^[57]^.

However, differences in the glycolytic metabolism of immune cells depend on a myriad of other factors besides the ones investigated in the current study, such as genetic predispositions or differences in microbiome ^[22–27]^, environmental factors such as diet ^[58]^, or previous encounters with certain pathogens ^[59,60]^. Likely, involvement of epigenetic factors plays an important role, as these are increasingly found to be regulated by metabolic pathways in immune cells ^[61–64]^. Although the distinct groups in healthy individuals display differences in glycolytic flexibility and functional responses of their immune cells, all variations likely fall within the adequate and healthy range of immune responses toward pathogens. Nevertheless, one might hypothesize that differences in disease susceptibility could be connected to the glycolytic response levels.

Diabetes mellitus is generally associated with an aberrant immune response leading to a chronic inflammatory state, but also an increased susceptibility to infections ^[28–32]^. In patients with diabetes mellitus that suffer from chronic hyperglycemia ^[65]^, immune cells are residing in a different extracellular environment that may play a pivotal role in determining both metabolic and inflammatory responses ^[66,67]^. Therefore, we investigated if the aforementioned relationships between lactate and cytokine production could be altered in patients with T1DM. Although many of the associations between lactate and specific cytokines could be reproduced, the association between IL-6 and lactate was significantly diminished. Furthermore, a decreased production of several cytokines was found relative to the production of lactate, compared with healthy individuals. These results are in accordance with earlier work, where a decreased association between glycolysis and cytokine production was found in monocytes of T1DM patients with a high glycemic burden ^[43]^. The frequent occurrence of hyperglycemic events in patients with T1DM could lead to increased glucose availability for immune cells. Subsequent increases in glucose utilization, without similar increases in cytokine secretion could be an explanation of the general attenuation that was seen for the association between lactate and cytokine production in this group. Especially the differential response to *S. aureus* could be disadvantageous for the host, and might relate to the higher frequency of infections that is often associated with the presence of diabetes. In the high-, intermediate-, and low-responding clusters that were identified based on the relative glycolytic flexibility, the distinct differences in response to *S. aureus* also seemed to be attenuated among the different clusters. Interestingly, however, cytokine responses to other PAMPs, including Pam3Cys and *C. albicans* showed an even more profound difference between the different groups of responders. This is intriguing, especially because patients with diabetes are often more susceptible to infection with *Candida* ^[68]^ and stimulation of monocytes from T1DM patients with Pam3Cys has revealed uncoupling of glycolysis and cytokine production before ^[43]^. It may be indicative of inefficient immune responses, particularly associated to the intermediate- or low-responding groups. Mechanistically, the altered responses might be explained by differences in cell surface expression of different TLRs or downstream signaling pathways due to the presence of diabetes that would ultimately translate into altered metabolic and functional responses.

In conclusion, we report that the production of lactate used as a marker for the glycolytic rate of innate immune cells correlates robustly with the production of several cytokines, across an array of different PAMPs. Therefore, in future cohort studies, lactate could be included as an easy and feasible additional measurement of innate immune cell activation and to determine glycolytic variability that exists between healthy individuals. Furthermore, future studies could focus on unraveling the possible causes and consequences of the high-, intermediate-, and low-responding groups and whether these clusters can be found consistently among other (metabolic) diseases. If beneficial effects could be attributed to any of these groups, elucidating the responsible host factors could lead to the identification of (epi)genetic targets of immune cell metabolism, which could be modulated to ultimately improve glycolytic responses and thus immune cell functioning.

## Author contributions

MGN, CJT, and RS designed the study. XAMHvD, FV, MJ, AWMJ, and AH performed the experiments. XAMHvD and FV analyzed the data. FV, XAMHvD, and RS wrote the paper with input from all authors.

## Conflicts of interest

The authors declare that they have no conflicts of interest.

## Funding

XAMHvD and FV were supported by a grant from the Cosun Nutrition Center. RS was supported by a VIDI-grant from the Dutch Research Council (NWO). The Human Functional Genomics Project is supported by an ERC Consolidator Grant (310372), a Spinoza grant (94-212) and IN-CONTROL CVON grant to MGN (CVON2012-03).

## Acknowledgments

We thank all the volunteers for their participation. The authors also thank Sanne Smeekens, Marije Doppenberg-Oosting, Clementine Verhulst, Lisa van de Wijer, Wouter van der Heijden and Lian van Meijel for their assistance during the setup of the cohorts; recruitment of study subjects or assistance during clinical visits. We thank Sanne Smeekens, Lisa van de Wijer, Wouter van der Heijden, Heidi Lemmers and Helga Dijkstra for assistance during experimental bench work; and Rob ter Horst for assistance during data processing.

## Supplementary Material


