# Supplementary material for: Glycolytic activity in human immune cells: inter-individual variation and functional implications during health and diabetes
Source: Immunometabolism (Cobham). 2022 Nov 1;4(4):e00008. doi: 10.1097/IN9.0000000000000008 (PMC9624385; doi:10.1097/IN9.0000000000000008)
Supplement: Supplementary file 1 [file in9-4-e00008-s001.pdf]

Supplemental Digital Content

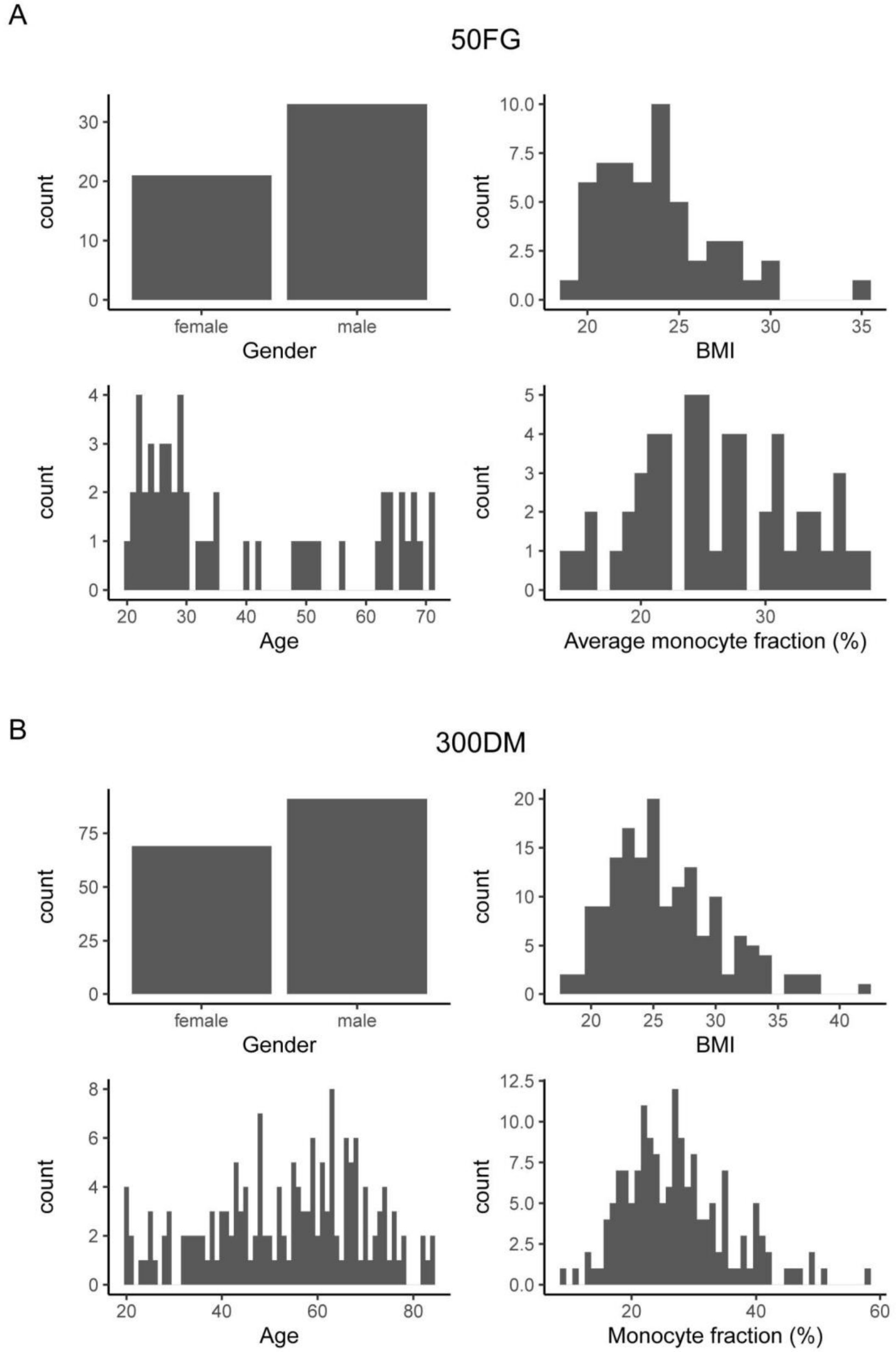

Figure S1. Subject characteristics of the 50FG (A) and 300DM (B) cohorts.

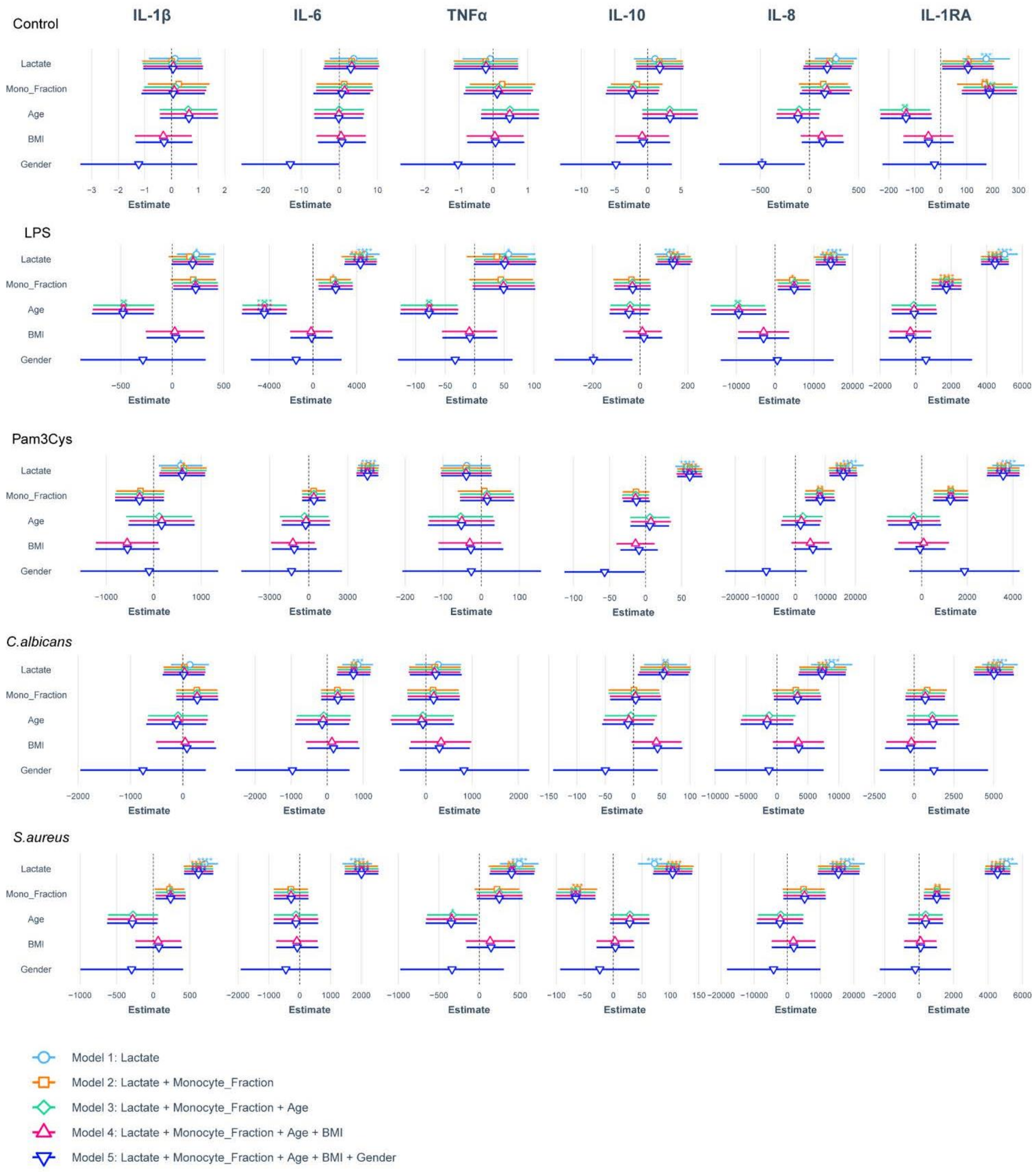

Figure S2. Separate mixed models were fitted for each of the cytokine-stimulation combinations. Host factors were added as covariates in a stepwise fashion to evaluate their individual impact on model parameters. All host factors were included as covariates in the final mixed model.

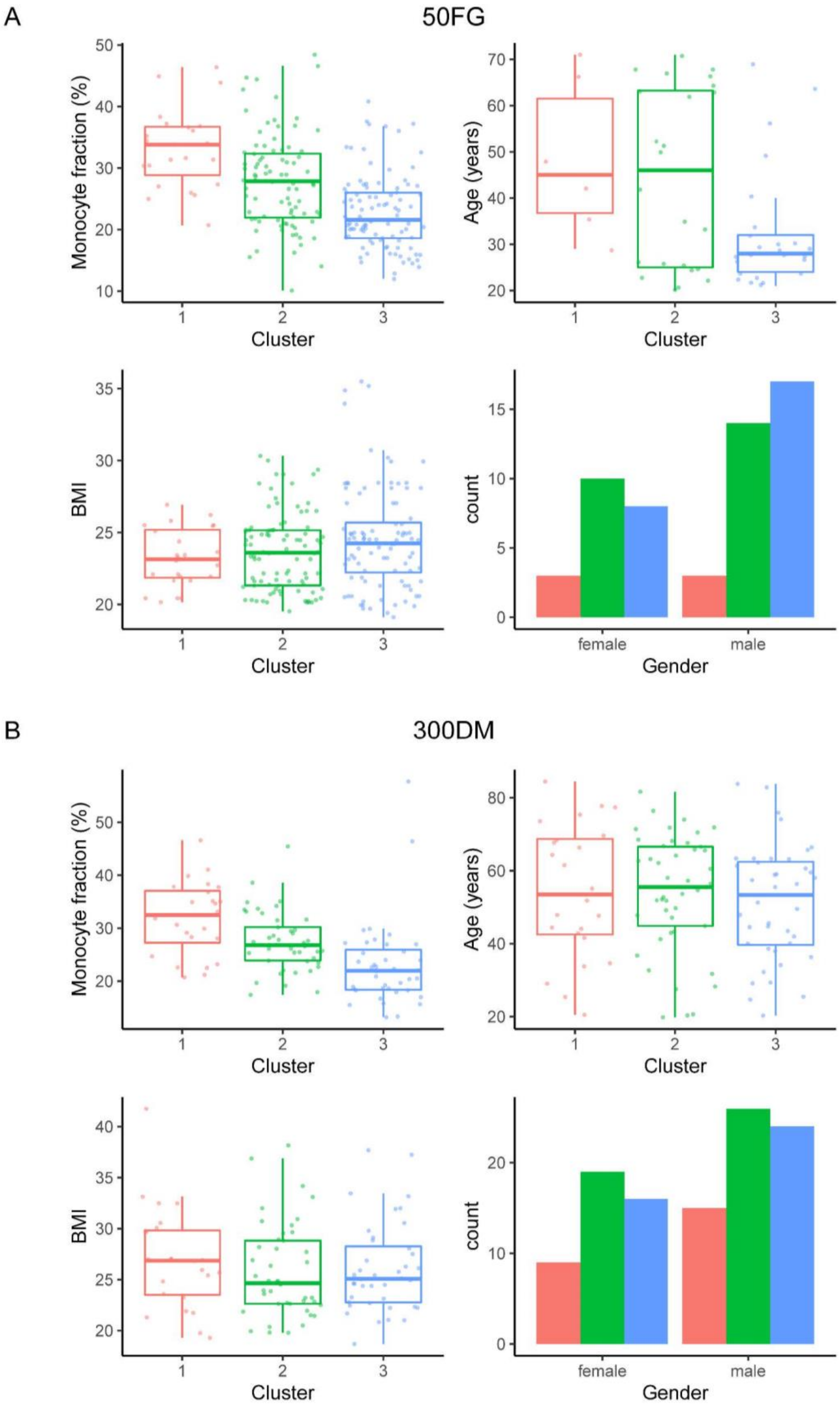

Figure S3. Univariate plotting of the host factors for each of the three clusters that were distinguished by k-means clustering for healthy subjects (A) and patients with T1DM (B). BMI: Body Mass Index.

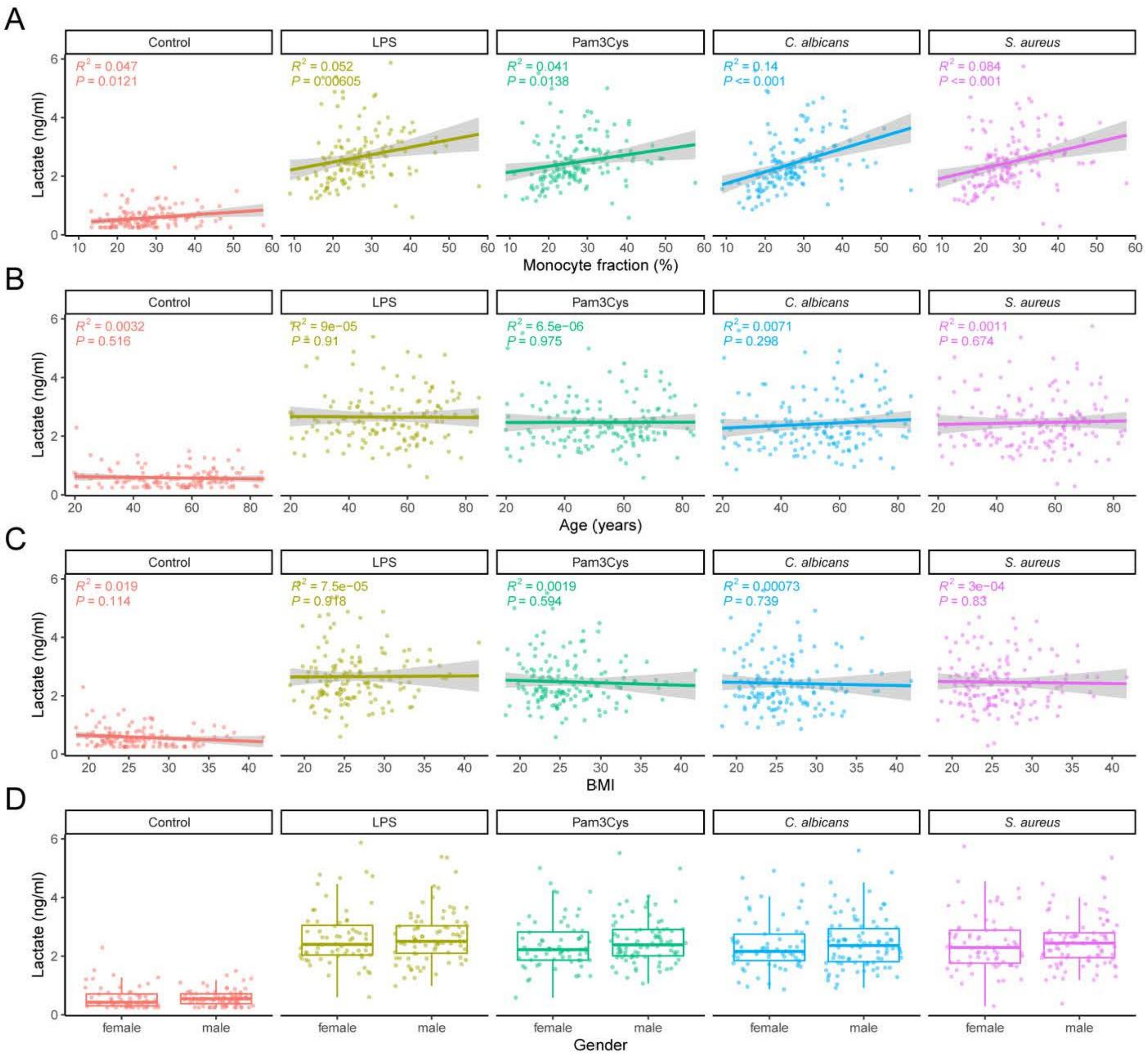

Figure S4. Linear relation between lactate production in PBMC's and monocyte fraction (A), age (B), BMI (C) and gender (D) for patients with T1DM. LPS: lipopolysaccharide; BMI: Body Mass Index.

Table S1. Overview of the p-value, false discovery rate (FDR) and the coefficient of determination (R^2) of all correlations between lactate and cytokines.

		50FG			300DM		
Cytokine	Stimulation	P-value	FDR	R^2	P-value	FDR	R^2
IL-1B	RPMI	0.842675	0.891714	0	0.397136	0.441262	0.005
IL-1B	LPS	0.411988	0.494385	0.003	0.856682	0.886223	0
IL-1B	Pam3Cys	0.001938	0.00342	0.066	0.01894	0.033423	0.037
IL-1B	*C. albicans conidia*	0.677183	0.752426	0.001	0.106069	0.159104	0.017
IL-1B	*S. aureus*	1.26E-05	2.92E-05	0.093	0.00026	0.0006	0.082
IL-6	RPMI	0.242056	0.30257	0.008	0.187208	0.255284	0.013
IL-6	LPS	1.55E-05	3.32E-05	0.092	4.29E-06	1.61E-05	0.138
IL-6	Pam3Cys	4.04E-17	2.43E-16	0.396	2.04E-11	1.23E-10	0.266
IL-6	*C. albicans conidia*	0.041382	0.053977	0.023	0.000107	0.000292	0.094
IL-6	*S. aureus*	2.72E-09	9.08E-09	0.166	0.000236	0.00059	0.083
TNFa	RPMI	0.861991	0.891714	0	0.602147	0.645158	0.002
TNFa	LPS	0.034042	0.04642	0.023	0.230576	0.292623	0.01
TNFa	Pam3Cys	0.01975	0.029625	0.038	0.394979	0.441262	0.005
TNFa	*C. albicans conidia*	0.646335	0.745771	0.001	0.948755	0.948755	0
TNFa	*S. aureus*	0.004286	0.006856	0.041	0.234098	0.292623	0.009
IL-10	RPMI	0.972171	0.972171	0	0.389749	0.441262	0.006
IL-10	LPS	0.000243	0.000486	0.068	0.000773	0.001546	0.076
IL-10	Pam3Cys	6.02E-11	2.26E-10	0.263	2.74E-06	1.17E-05	0.14
IL-10	*C. albicans conidia*	0.004342	0.006856	0.045	0.000489	0.001049	0.077
IL-10	*S. aureus*	4.49E-06	1.12E-05	0.103	0.052674	0.087791	0.024
IL-8	RPMI	0.023908	0.034154	0.028	0.068183	0.107658	0.025
IL-8	LPS	3.54E-08	9.66E-08	0.146	1.63E-05	5.42E-05	0.122
IL-8	Pam3Cys	1.15E-11	5.75E-11	0.279	3.15E-08	1.57E-07	0.19
IL-8	*C. albicans conidia*	7.53E-09	2.26E-08	0.172	0.000104	0.000292	0.094
IL-8	*S. aureus*	5.45E-11	2.26E-10	0.198	0.002163	0.004056	0.059
IL-1RA	RPMI	0.000461	0.000865	0.067	0.120116	0.171594	0.018
IL-1RA	LPS	1.69E-27	2.54E-26	0.458	1.50E-15	2.25E-14	0.36
IL-1RA	Pam3Cys	3.90E-24	3.90E-23	0.518	4.80E-14	4.80E-13	0.323
IL-1RA	*C. albicans conidia*	2.16E-20	1.62E-19	0.384	1.58E-12	1.18E-11	0.279
IL-1RA	*S. aureus*	1.42E-37	4.26E-36	0.57	1.18E-17	3.53E-16	0.375
